# Multifactorial Risk Assessment of Falls in Thai Community-Dwelling Older Adults: Findings from a Geriatric Cohort Study

**DOI:** 10.3390/geriatrics10050118

**Published:** 2025-09-03

**Authors:** Natthaphon Ubonsutvanich, Aisawan Petchlorlian, Bhorn-Ake Manasvanich, Rapas Samalapa, Thanyaporn Hengpongthorn, Jirapa Champaiboon, Kaewkanda Lekmanee, Seangarun Surawong, Kearkiat Praditpornsilpa

**Affiliations:** 1 Geriatric Excellence Centre, King Chulalongkorn Memorial Hospital, Thai Red Cross Society, Bangkok 10330, Thailand; ubo.natthaphon@gmail.com (N.U.);; 2 Division of Geriatric Medicine, Department of Medicine, Faculty of Medicine, Chulalongkorn University, Bangkok 10330, Thailand; 3 Department of Family Medicine, King Chulalongkorn Memorial Hospital, Thai Red Cross Society, Bangkok 10330, Thailand; 4 Department of Rehabilitation Medicine, Faculty of Medicine, Chulalongkorn University, Bangkok 10330, Thailand; 5 Department of Rehabilitation Medicine, King Chulalongkorn Memorial Hospital, Thai Red Cross Society, Bangkok 10330, Thailand; 6 Department of Nursing, King Chulalongkorn Memorial Hospital, Bangkok 10330, Thailand; 7 Department of Medicine, Faculty of Medicine, Chulalongkorn University, Bangkok 10330, Thailand

**Keywords:** falls, risk factors, older adults

## Abstract

**Background/Objectives**: Falls are a major public health concern among older adults, often resulting in injury, mortality, and loss of independence. Understanding fall-related risk factors is essential for developing effective prevention strategies. This study examined the multifactorial risk assessment of falls among Thai community-dwelling older adults, aiming to identify and prioritize modifiable risk factors for targeted interventions in the Thai context. **Methods**: A cross-sectional study was conducted among 5694 adults aged ≥60 years who attended a comprehensive geriatric clinic in Bangkok, Thailand, between March 2019 and December 2023. All participants underwent a comprehensive geriatric assessment and fall history screening. Logistic regression analysis was performed to identify independent predictors of falls and recurrent falls. **Results**: Among the 5694 participants, 17.7% reported at least one fall in the past year, and 4.1% experienced recurrent falls. Independent risk factors for falls included female sex (OR = 1.74), unsteadiness (OR = 1.54), fear of falling (OR = 1.22), sedative drug use (OR = 1.38), and low gait speed (<1 m/s; OR = 1.70). Recurrent falls were additionally associated with urinary incontinence (OR = 1.78). Most falls occurred outdoors (58.5%), primarily due to environmental hazards such as slippery floors and uneven surfaces. The Clinical Test of Sensory Integration of Balance (CTSIB) showed no difference between fallers and non-fallers, except under the eyes-open on firm surface condition, where recurrent fallers exhibited significantly greater postural sway (*p* = 0.048). **Conclusions**: In community-dwelling Thai older adults with robust or pre-frail status, the three key questions for fall risk screening appear to be the most effective tool. Modifiable risk factors strongly associated with fallers and recurrent fallers include sedative use, urinary incontinence, and unsteadiness. Accordingly, medication review, urinary incontinence screening, and balance assessment may help prevent falls. The CTSIB may have only limited value in differentiating fall risk between fallers and non-fallers in this population.

## 1. Introduction

Thailand is undergoing a demographic transition into a fully aged society. As individuals age, the risk of falling increases significantly, making falls a critical public health concern among older adults. Falls are a common geriatric syndrome, affecting approximately one in four older adults annually [1]. However, many falls go unreported—particularly among so-called “silent fallers” who do not seek medical attention unless an injury occurs. A fall is defined as an event in which an individual unintentionally comes to rest on the ground, floor, or a lower level [2].

In Thailand, the prevalence of falls among older adults ranges from 12% to 22% [3,4,5]. Among individuals aged ≥65 years, falls account for 11% of all-cause mortality [6], and the prevalence of fear of falling is 35.8% [3]. Nationwide screening by the Ministry of Public Health identified 55.6% of community-dwelling older adults as being at high risk for falls [5].

The risk of falling is multifactorial, involving intrinsic factors (e.g., age-related declines in muscle strength, balance, vision, hearing, cognitive function, sarcopenia, and gait disturbances), behavioral factors (e.g., medication use, alcohol consumption, sedentary lifestyle, and reduced balance confidence), socio-economic and psychosocial factors (e.g., low education, social isolation, depression, and anxiety), and environmental hazards (e.g., slippery surfaces, uneven pathways, poor lighting, and unsafe footwear) [7,8,9].

Balance impairment is one of the most frequent contributors to falls in older adults [10]. Balance relies on the integration of sensory inputs from proprioceptors, vision, and the vestibular system [11]. With aging, proprioceptive sensitivity diminishes, the density of cutaneous receptors in the lower limbs decreases, and vibratory thresholds increase, all of which contribute to postural instability [12]. The Clinical Test of Sensory Integration of Balance (CTSIB) provides a useful tool for evaluating these sensory components and identifying specific deficits for targeted intervention.

Our study aims to identify risk factors for falls among older adults in Thailand using comprehensive geriatric assessments. In contrast to previous Thai studies that focused primarily on intrinsic determinants, the present investigation incorporates both intrinsic factors (e.g., sarcopenia, balance performance) and extrinsic factors (e.g., slippery floors, uneven surfaces, poor lighting, improper footwear) [4,9], while also drawing upon a substantially larger sample size. This dual-domain assessment offers a more nuanced and culturally relevant characterization of fall risk profiles in community-dwelling older adults, integrating health-related conditions with environmental challenges.

These insights may support the development of fall-risk assessment tools and inform proactive interventions to prevent falls in this population.

## 2. Materials and Methods

### 2.1. Study Design, Setting, and Participants

This cross-sectional study was conducted within an ongoing geriatric cohort at a university hospital in Bangkok, Thailand. Adults aged ≥60 years who met the inclusion criteria and attended the geriatric outpatient clinic for a standardized Comprehensive Geriatric Assessment (CGA) and routine health check-ups between March 2019 and December 2023 were eligible. All patients underwent a standardized CGA, and those reporting a fall received a detailed interview and additional fall risk assessments. For analysis, only data from each patient’s first visit were included, resulting in a cross-sectional dataset.

Inclusion criteria required participants to be aged 60 years or older and functionally independent in basic activities of daily living. Exclusion criteria included: active malignancy or a history of malignant disease within one year of completed treatment; recent hospitalization (within the past three months) for cardiac, pulmonary, or neurovascular conditions; chronic kidney disease requiring renal replacement therapy; and active psychiatric disorders requiring medication adjustment within the past three months.

### 2.2. Fall History, Screening, and Classification

Participants were interviewed face-to-face by trained geriatric nurses using a web-based data collection system. Demographic data were collected, including age, sex, marital status, education level, residential area, living situation (e.g., living alone), underlying medical conditions, and the number of medications. Participants were asked three screening questions adapted from the Stopping Elderly Accidents, Deaths & Injuries (STEADI) fall-risk screening algorithm. The Thai version was adapted from the original U.S. CDC STEADI program through forward–backward translation and cultural adaptation. It demonstrated good validity (IOC: 0.80–1.00) and reliability (Cronbach’s α = 0.78; ICC = 0.95) [13,14].

Have you fallen in the past year?

Do you feel unsteady when walking?

Do you have a fear of falling?

For those who reported a fall, additional details were obtained, including the location, time, pre- and post-fall symptoms, injuries sustained, and environmental factors involved. A fall was defined as “an event that results in a person inadvertently coming to rest on the ground, floor, or another lower level.” Participants reporting two or more falls within a 12-month period were classified as recurrent fallers [15].

### 2.3. Mobility Assessment

Mobility was assessed using gait speed and the Timed Up and Go (TUG) test [16]. Gait speed was measured over a 6 m walk at the participant’s usual pace using a stopwatch. Two trials were conducted, and the average time was converted into meters per second (m/s) [17].

### 2.4. Balance Assessment

Balance was assessed using the modified CTSIB with the Biodex Balance System SD (Biodex Medical Systems, Inc., Shirley, NY, USA), administered by a physiotherapist according to the system’s standardized protocol [18,19]. The Biodex Balance System SD incorporates the Balance Error Scoring System (BESS), which has demonstrated strong criterion validity through correlations with laboratory-based balance measures and established construct validity [18,20].

The test assesses the integration of somatosensory, visual, and vestibular input under four conditions: firm surface, eyes open (baseline), firm surface, eyes closed (somatosensory input), foam surface, eyes open (visual and vestibular input), and foam surface, eyes closed (vestibular input). Each condition was tested for 30 s. The test was stopped if the participant moved their arms or feet. If a participant could not maintain the position for 30 s, up to two additional attempts were allowed. The sway index from the three trials was averaged and compared with normative data for individuals aged 65–84 years (Table 1) [21].

### 2.5. Comprehensive Geriatric Assessment (CGA)

Participants underwent a CGA that included evaluation of multiple geriatric syndromes. Nutritional status was assessed using the Mini Nutritional Assessment (MNA) [22] and Body Mass Index (BMI). Polypharmacy was determined based on the number of medications. Urinary incontinence was assessed by self-report. Frailty was identified using the frailty phenotype [23]. Cognitive function was assessed using the Montreal Cognitive Assessment (MoCA) [24]. Sarcopenia was diagnosed according to the 2019 criteria of the Asian Working Group for Sarcopenia [25].

Functional abilities were evaluated using two scales: the Barthel Index for Basic Activities of Daily Living (BADL) [26] and the Lawton Instrumental Activities of Daily Living (IADL) Scale [27].

### 2.6. Identification of Fall Risk Factors

Multifactorial fall risk factors were identified using the U.S. Centers for Disease Control and Prevention (CDC) Algorithm for Fall Risk Screening, Assessment, and Intervention [28], supported by evidence from international meta-analyses [29] and Thai studies [9]. Based on these sources, the CGA included age, sex, fear of falling, unsteadiness, low muscle mass, cognitive impairment, polypharmacy, and urinary incontinence.

### 2.7. Ethical Considerations

The institutional review board approved the cohort study (date of first registration: 25 October 2019, IRB approval no. 718/62). The study was registered as a clinical trial on 10 May 2020, with the identifier number TCTR20200511003. Before participating in the study, all participants provided written informed consent. This study is reported following the STROBE guidelines [30].

Language polishing and grammar correction were supported by ChatGPT (OpenAI, version 5.0.1.0, San Francisco, CA, USA), an AI-based language model. The tool was used exclusively to assist with sentence structure refinement and grammatical accuracy. The authors reviewed and verified all AI-assisted modifications to ensure accuracy and integrity of the content.

### 2.8. Statistical Analysis

Data were analyzed using IBM SPSS Statistics version 29.0.1.0. The overall prevalence of falls was first calculated. Participant characteristics were summarized by fall and non-fall groups as mean ± standard deviation (SD). Bivariate analyses were conducted to identify potential factors associated with falls. Given the dichotomous nature of the dependent variable, logistic regression was used to estimate crude odds ratios (ORs) as measures of association. Adjusted ORs and their 95% confidence intervals (CIs) were obtained by including all selected independent variables in a multivariable logistic regression model. Differences in the sway index from the CTSIB between fall and non-fall groups were evaluated using independent *t*-tests. Statistical significance was set at *p* < 0.05.

## 3. Results

### 3.1. Baseline Data of Participants

Among the 5694 participants, the majority were classified as robust (55.6%), followed by pre-frail (42.9%) and frail (1.5%) (Table 2). The mean age was similar across groups, although slightly lower in the frail group (66.22 ± 4.69 years). Females comprised 74.5% of the total sample, with similar proportions across all frailty categories.

The overall prevalence of falls in the total sample was 17.7%, with the highest rate observed among robust participants (19.1%), and followed by the pre-frail (15.9%) and frail groups (15.7%). Recurrent falls were reported by 4.1% of all participants, showing a similar pattern: 5.1% in the robust, 4.3% in the pre-frail, and 1.4% in the frail group. The prevalence of unsteadiness and fear of falling was relatively consistent across all frailty categories, at approximately 17% and 26%, respectively. Most baseline characteristics were comparable across the three frailty groups, with no clinically significant differences observed, except for age, which was slightly lower in the frail group (Table 2).

### 3.2. Characteristics of Fall Events and Injuries

A total of 2322 fall events were reported, with the majority occurring outdoors (58.5%). Environmental hazards such as slippery floors and uneven surfaces were the leading contributors, followed by inappropriate footwear and poor lighting. Among 1704 fall-related injuries, most were minor; however, 8.6% were serious, including fractures of the lower and upper extremities, hips, and spine, as well as rare cases of loss of consciousness and intracranial bleeding (Table 3).

### 3.3. Risk Factors Associated with Falls and Recurrent Falls

Of the 5694 participants, 17.7% reported at least one fall in the past year and 4.1% experienced recurrent falls. Logistic regression analysis showed that female sex, unsteadiness, fear of falling, sedative drug use, and low gait speed were significantly associated with increased odds of falling, while pre-frailty status was inversely associated with fall risk. For recurrent falls, female sex remained the strongest predictor, together with unsteadiness, fear of falling, sedative use, and urinary incontinence. Other variables, including age, polypharmacy, sarcopenia, and cognitive function, were not significantly associated with fall risk (Table 4 and Table 5).

### 3.4. CTSIB Assessment

Sensory and balance functions were assessed using the modified CTSIB under four sensory conditions (Table 6). Sway indices were generally similar between fallers and non-fallers across most conditions. However, under the baseline condition (eyes open on a firm surface), recurrent fallers exhibited significantly greater sway than non-fallers (mean = 1.23 vs. 1.13 mm/s; *p* = 0.048), suggesting age-related postural instability even in the simplest balance setting. In the condition with eyes closed on a firm surface, which primarily assesses somatosensory function, both fallers and recurrent fallers showed a trend toward increased sway, although the differences did not reach statistical significance. No significant differences were observed in the remaining conditions among fallers, recurrent fallers, and non-fallers.

However, when comparing the sway indices of all participants with normative data from independent adults aged 65–84 years in the United States (*n* = 215) [22], we found that in both the eyes-open and eyes-closed conditions on a foam surface, the results exceeded 2 standard deviations in both fallers and non-fallers. This suggests that the study population may have vestibular-related impairments in their balance system.

## 4. Discussion

This study revealed a prevalence of falls (17.7%) and recurrent falls (4.1%) among community-dwelling healthy older adults, with most incidents occurring outdoors. Interestingly, robust individuals reported more falls than frail older adults, possibly due to greater engagement in outdoor or physical activities. Although physical and cognitive performance was comparable across frailty groups, robust and pre-frail participants may have had greater exposure to environmental hazards, particularly when mobility is preserved but attentional awareness may be diminished. This is consistent with the finding that most falls were attributed to extrinsic factors, such as slippery and irregular surfaces.

The prevalence of falls in this study, estimated at approximately 20%, is comparable to that reported in other studies with a similar mean age. However, studies from Singapore and Europe, which included populations aged ≥40 years and ≥55 years, respectively, reported lower fall prevalence. The prevalence of falls in most studies was based on the question: ‘Have you fallen in the past year?’ This may be a limitation in older adults, as it relies on self-reporting and may be affected by recall bias, which could contribute to variability in fall prevalence across studies.

Our study identified risk factors significantly associated with falls, including female sex, unsteadiness, and sedative use—factors commonly implicated in the multifactorial nature of fall risk among older adults [31]. Risk factors with borderline statistical significance, namely low gait speed and fear of falling, indicate weak associations and should be interpreted with caution and validated in future studies.

Notably, a distinctive finding in our study was that pre-frailty was associated with a lower risk of falling (OR 0.8), possibly reflecting more cautious behavior and a higher level of awareness regarding fall prevention among participants. However, some commonly recognized risk factors, such as sarcopenia and high Timed Up and Go (TUG) test scores, were not found to be significant. This may be attributed to the fact that the majority of participants were classified as robust or pre-frail and retained good physical performance and muscle strength.

In Thailand, cultural and environmental factors likely influence fall risk among older adults. Urban older adults report poorer general health than their rural counterparts [32] and rely more on walking and public transport. In Bangkok, 56.3% of elderly residents use buses and rail transit, while private car use remains comparatively low [33]. However, only about 20% perceive their neighborhoods as suitable for walking [34], reflecting limited age-friendly infrastructure. These inadequacies increase exposure to uneven or slippery terrain and help explain the higher prevalence of falls in urban areas [32]. While recent studies have focused mainly on modifiable in-home hazards [4], fall prevention for active older adults should also emphasize strengthening intrinsic factors and promoting outdoor safety. Since outdoor environmental factors are difficult to modify at the individual level, city-wide and governmental policies are essential to create safer and more age-friendly environments.

Table 7 presents the risk factors for falls identified in our study compared with large cohorts from countries with established geriatric societies. Some risk factors, such as female sex and urinary incontinence, were consistently observed across multiple cohorts, including the SEED study in Singapore, the SHARE study in Europe, and a large-scale study from the United States. However, several high-prevalence risk factors reported in international literature—such as advanced age, frailty, and polypharmacy—were not found to be significant in our study. This may be explained by the relatively healthier sample, in which most participants were classified as robust or pre-frail. Additionally, fear of falling showed a modest effect size in the Thai context (OR 1.22), in contrast to a much stronger association reported in European data (OR 3.75), possibly reflecting differences in perception, reporting, or fall prevention awareness among older adults across cultural settings.

We found that commonly used tools for fall risk screening showed minimal differences in mean TUG time between fallers and non-fallers. The overall average TUG time was 9.23 ± 1.80 s. Applying the World Falls Guidelines [40] cutoff of TUG > 15 s, only 2.68% of fallers screened positive, suggesting that TUG may not be suitable as a standalone screening tool in this population. In contrast, the use of the three key screening questions from the STEADI algorithm [7,14] appears more appropriate, as 34.56% of fallers reported a fear of falling and 26.42% reported feeling unsteady while walking. These findings align with a prospective study showing that the three key STEADI questions predicted falls within 12 months, with a sensitivity of 93.9% and specificity of 75% [41].

In this study, some participants underwent balance assessment using the CTSIB, which showed no significant difference between fallers and non-fallers. In the CTSIB comparison, only a borderline significant difference in the baseline condition was observed between recurrent fallers and non-fallers. This may be attributed to the fact that most participants were robust or pre-frail, physically active, and able to go outdoors independently, as reflected by the predominance of outdoor falls. In such robust individuals, CTSIB did not reveal notable differences in sensory or balance function compared with the general population, suggesting that CTSIB may have limited value for fall risk assessment in this population. Recurrent fallers exhibited significantly greater postural sway than non-fallers under the simplest CTSIB condition (eyes open on a firm surface), suggesting that even basic balance tasks can reveal subtle postural control deficits. This supports the clinical relevance of including the 4-stage balance test in fall risk assessment. Consistent with previous studies, extending the stance duration beyond 10 s may improve prediction of falls within six months in older adults who appear otherwise robust [42]. The most common problems identified by CTSIB were vestibular and visual dysfunction. These findings suggest that older adults should participate in vestibular and balance training exercises and undergo an annual ophthalmologic examination. CTSIB may be more appropriate for independent frail older adults and for recurrent fallers with predominantly indoor falls, as it can help evaluate specific components of balance impairment.

A major strength of this study is its large sample size of 5694 community-dwelling older adults and the application of comprehensive geriatric assessments. The inclusion of diverse domains—encompassing physical function, sensory integration assessed by CTSIB, psychological status, and environmental exposures—provides a holistic perspective on fall risk factors.

However, this study has several limitations. First, fall events were self-reported, which may introduce recall bias, especially among participants with subtle cognitive decline or those reluctant to report falls due to stigma or forgetfulness. Second, although comprehensive geriatric assessments were conducted, detailed classifications of underlying conditions such as specific cardiovascular, neurological, or musculoskeletal diseases were not captured, limiting the ability to account for their potential influence on fall risk. Third, although the cross-sectional design limits causal inference, the identified risk factors may represent common contributors to falls. These findings can inform preliminary prevention strategies and guide the selection of candidate factors for prospective cohort studies to confirm causality. Longitudinal data from such studies could further clarify the underlying mechanisms of falls, strengthening the evidence base for targeted interventions.

This study identified multifactorial risk factors, including intrinsic factors, unsteadiness, fear of falling, sedative use, low gait speed, and urinary incontinence and extrinsic factors, which are important given that independent older adults often experience falls in outdoor environments. These findings highlight the need for a comprehensive fall risk assessment encompassing three domains: (1) physical and geriatric syndromes, with attention to medication-related falls, gait speed, urinary incontinence, and unsteadiness requiring further balance evaluation; (2) psychological factors, particularly fear of falling; and (3) environmental hazards in the surrounding context. Incorporating the CTSIB into routine assessments may help detect subtle sensory deficits in independent older adults and may be especially beneficial for frail individuals. Recognizing that most falls occur in specific environmental contexts can inform targeted education and home safety interventions, supporting a multifaceted approach to fall prevention.

## 5. Conclusions

In community-dwelling Thai older adults with robust or pre-frail status, the three key questions for fall risk screening appear to be the most effective tool. Modifiable risk factors strongly associated with fallers and recurrent fallers include sedative use, urinary incontinence, and unsteadiness. Accordingly, medication review, urinary incontinence screening, and balance assessment may help prevent falls. The CTSIB appears to have limited utility in distinguishing fall risk between fallers and non-fallers in this population. These findings suggest that older adults should engage in vestibular and balance training exercises and undergo annual ophthalmologic examinations. The CTSIB may be more suitable for independent frail older adults and for recurrent fallers with predominantly indoor falls, as it can help assess specific components of balance impairment.

## Figures and Tables

**Table 1:** The CTSIB normative data in older adults aged 65–84 years [21].

Conditions	Sway Indices Mean (SD): (mm/s)
Eyes open, firm surface	0.66 (0.40)
Eyes closed, firm surface	1.17 (0.38)
Eye open, foam surface	1.13 (0.38)
Eye closed, foam surface	3.50 (0.32)

**Table 2:** Baseline Characteristics of Participants by Frailty Status.

	Total *n* = 5694 (100%)	Robust *n* = 3169 (55.65%)	Pre-Frailty *n* = 2442, (42.89%)	Frailty *n* = 83, (1.46%)	*p*-Value
● Age (year, mean ± SD)	67.48 (5.15)	67.41 (5.13)	67.61 (5.23)	66.22 (4.69)	0.029
● Sex (Female) (%)	4243 (74.5%)	2354 (74.3%)	1829 (74.9%)	60 (72.3%)	0.781
● Fall (%)	1007 (17.7%)	605 (19.1%)	389 (15.9%)	13 (15.7%)	0.008
● Recurrent Fall (%)	233 (4.1%)	139 (5.1%)	93 (4.3%)	1 (1.4%)	0.227
● Unsteadiness * (yes, %)	981 (17.2%)	548 (17.3%)	424 (17.4%)	9 (10.8%)	0.299
● Fear of Falling * (yes, %)	1475 (25.9%)	821 (25.9%)	637 (26.1%)	17 (20.5%)	0.519
● Polypharmacy (%)	2363 (41.5%)	1321 (41.7%)	1017 (41.6%)	25 (30.1%)	0.106
● Sedative drugs use (%)	546 (9.6%)	290 (9.2%)	252 (10.3%)	4 (4.8%)	0.112
● Exercise < 150 min/week (%)	2079 (36.5%)	1172 (43.0%)	877 (42.0%)	30 (42.9%)	0.709
● Urinary Incontinence (%)	1428 (25.1%)	798 (25.2%)	607 (24.9%)	23 (27.7%)	0.824
● Low muscle mass [25] (%)	2382 (41.8%)	1300 (41.0%)	1055 (43.2%)	27 (32.5%)	0.056
● Sarcopenia [25] (%)	777 (13.6%)	438 (13.8%)	332 (13.6%)	7 (8.4%)	0.368
● Gait Speed (m/s, Mean ± SD)	1.46 (0.20)	1.46 (0.20)	1.46 (0.21)	1.48 (0.18)	0.675
● TUG (s, Mean ± SD)	9.23 (1.80)	9.21 (1.85)	9.29 (1.90)	9.05 (1.33)	0.180
● MoCA score (Mean ± SD)	26.01 (2.85)	26.01 (2.80)	25.94 (2.86)	26.36 (2.94)	0.312

* A positive response to a question from the STEADI fall screening [13].

**Table 3:** Summary of Fall Events, Contributing Factors, and Injuries: Total fall events 2322 times.

Location of Fall	Contributing Fall Risk Factors	Fall-Related Injury Types (1704 Events)
Home (41.5%) Outdoors (58.5%)	Slippery floor (40.4%) Uneven surface/steps (38.0%) Improper footwear (9.00%) Poor lighting (4.2%) Loose clothing (0.9%) Visual impairment (1.63%)	Serious injury (8.57%) - Lower extremities fracture (3.25%) - Upper extremities fracture (3.37%) - Hip fracture (0.81%) - Spinal fracture (0.52%) - Loss of consciousness (0.58%) - Intracranial bleeding (0.04%) Minor injury (91.43%)

**Table 4:** Comparison of Participant Characteristics and Fall Risk Factors between Fallers and Non-Fallers Baseline Characteristics of Participants by Frailty Status.

	Non-Fallers (*n* = 4687)	Fallers (*n* = 1007)	OR (95%CI)	*p*-Value
○ Age (year, mean ± SD)	67.38 (5.07)	67.95 (5.60)	1.00 (0.99, 1.02)	0.785
○ Age (year, mean ± SD)				
- Female (%)	3408 (72.7%)	835(82.9%)	1.74 (1.29, 2.36)	<0.001
- Male (%)	1279 (27.3%)	172 (17.1%)	Reference	
○ Waist circumference (cm): mean ± SD	82.10 (9.76)	82.48 (10.07)	1.02 (1.00, 1.03)	0.030
○ Unsteadiness * (yes, %)	715 (15.3%)	266 (26.4%)	1.54 (1.26, 1.88)	<0.001
○ Fear of falling * (yes, %)	1127 (24%)	348 (34.6%)	1.22 (1.02, 1.45)	0.029
○ Exercise < 150 min/week (%)	1710 (36.5%)	369 (36.6%)	0.95 (0.81, 1.11)	0.506
○ Polypharmacy (%)	1894 (40.4%)	469 (46.6%)	1.10 (0.94, 1.29)	0.256
○ Sedative drugs use (%)	410 (8.7%)	136 (13.5%)	1.38 (1.09, 1.75)	0.009
○ BADL score (mean ± SD)	99.49 (1.66)	99.24 (2.08)	0.98 (0.94, 1.02)	0.360
○ IADL scale (mean ± SD)	7.53 (0.82)	7.57 (0.79)	1.01 (0.91, 1.12)	0.900
○ Gait speed (m/s, mean ± SD)	1.47 (0.20)	1.42 (0.22)	0.81 (0.48, 1.36)	0.420
○ Low gait speed < 1 m/s (%)	112 (2.4%)	61 (6.1%)	1.70 (1.03, 2.81)	0.040
○ TUG test (s, mean ± SD)	9.19 (1.79)	9.48 (2.16)	0.97 (0.92, 1.03)	0.280
○ Low muscle mass [25] (%)	1924 (41%)	458 (45.5%)	1.12 (0.90, 1.40)	0.330
○ Sarcopenia [25] (%)	618 (13.2%)	159 (15.8%)	0.78 (0.55, 1.10)	0.160
○ MoCA score (mean ± SD)	26.01 (2.80)	25.88 (2.95)	0.99 (0.97, 1.03)	0.930
○ Urinary incontinence (%)	1113 (23.7%)	315 (31.3%)	1.14 (0.96, 1.37)	0.140
○ Frailty				
Pre-frail (%)	2053 (43.8%)	389 (38.6%)	0.80 (0.68, 0.93)	0.040
Frail (%)	80 (1.5%)	13 (1.3%)	0.98 (0.52, 1.87)	0.960

* A positive response to a question from the STEADI fall screening [13].

**Table 5:** Odds Ratios of Risk Factors of Recurrent Falls in Older Adults Compared to Non-Fallers.

	Non-Fallers (*n* = 4687)	Recurrent Fallers (*n* = 233)	OR (95%CI)	*p*-Value
○ Age (year, mean ± SD)	67.38 (5.07)	68.18 (5.63)	1.01 (0.98, 1.04)	0.730
○ Gender				
- Female (%)	3408(72.7%)	209 (89.7%)	4.83 (2.51, 9.27)	<0.001
- Male (%)	1279(27.3%)	24 (10.3%)	Reference	
○ Unsteadiness * (yes, %)	715 (15.3%)	88 (37.8%)	2.24 (1.59, 3.16)	<0.001
○ Fear of falling * (yes, %)	1127 (24%)	108 (46.4%)	1.53 (1.11, 2.11)	0.009
○ Sedative drugs use (%)	410 (8.7%)	46 (19.7%)	1.98 (1.34, 2.92)	<0.001
○ Urinary incontinence (%)	1113 (23.7%)	98 (42.1%)	1.78(1.30, 2.44)	<0.001

* A positive response to a question from the STEADI fall screening [13].

**Table 6:** Postural Sway Indices by CTSIB Under Different Sensory Conditions among Non-Fallers (*n* = 555), Fallers (*n* = 353), and Recurrent Fallers (*n* = 118).

		Mean (SD): (mm/s)	*p*-Value
○ Sway indices Condition: Eyes open, firm surface	Non-fallers	1.13 (0.04)	NA
	Fallers	1.16 (0.40)	0.232
	Recurrent fallers	1.23 (0.46)	0.048
○ Sway indices Condition: Eyes closed, firm surface	Non-fallers	1.63 (0.61)	NA
	Fallers	1.69 (0.58)	0.150
	Recurrent fallers	1.77 (0.64)	0.269
○ Sway indices Condition: Eyes open, foam surface	Non-fallers	2.36 (0.71)	NA
	Fallers	2.34 (0.73)	0.632
	Recurrent fallers	2.39 (0.84)	0.970
○ Sway indices Condition: Eyes closed, foam surface	Non-fallers	5.06 (1.44)	NA
	Fallers	4.99 (1.44)	0.428
	Recurrent fallers	5.10 (1.36)	0.937

NA = not applicable; non-fallers were used as the reference group.

**Table 7:** Comparison of Fall Prevalence and Associated Risk Factors Among Older Adults Across Previous Studies.

Study	Prevalence of Fall	Risk Factors (OR)
○ The Singapore Epidemiology of Eye Diseases (SEED) Study [35] (*n* = 10,009; mean [SD] age = 58.9 [10.4] years, community-dwelling adults aged ≥40 years)	○ 13.8% falls ○ 4.6% recurrent	- Female (1.79) - Older age (per decade increase) (1.20) - ≥3 comorbidities (1.35) - Visual impairment (VA > 20/60 in better eye) (1.23) - Alcohol consumption (1.41)
○ Guangzhou Study, China [36] (*n* = 1629; mean [SD] age = 69.5 [6.9] years; community-dwelling older adults aged ≥65 years)	○ 21.6%	- Female (1.721) - Age ≥ 80 years (1.910) - High-intensity physical activity (2.254) - ≥3 comorbidities (1.930) - Medium-intensity physical activity (0.721)
○ English Longitudinal Study of Ageing (ELSA) [37] (*n* = 4301; mean [SD] age = 74.3 [6.9] years, frailty 13.%)	○ 8.2%	- Severe pain (1.90) - Incontinence (1.48) - Frailty (1.69) - 2 comorbidities (1.33)
○ The Survey of Health, Ageing and Retirement in Europe (SHARE) [38] (*n* = 41,098; mean [SD] age = 70.0 [8.9] years, frailty 9.9%)	○ 8.2%	- Age ≥ 85 years (2.01) - Female (1.31) - Polypharmacy (1.42) - Pre-frail status (1.33) - Frail status (2.11) - Fear of falling (3.75)
○ Risk Assessment and Prevention of Falls in Older Community-Dwelling Adults in the US [39] (*n* = 13,685,662, age ≥ 65 years)	○ 27.5%	- Cognitive impairment (>2) - Gait abnormality (>2) - Arthritis (>1.5) - Frailty (>1.5) - Orthostatic hypotension (>1.5) - Urinary incontinence (>1.5) - Visual impairment (>1.5) - Pain (>1.5)

## Data Availability

The dataset produced and/or analyzed during the current study can be obtained from the corresponding author upon reasonable inquiry.

## Abbreviations

The following abbreviations are used in this manuscript:

CTSIB	Clinical Test of Sensory Integration of Balance
STEADI	Stopping Elderly Accidents, Deaths & Injuries
TUG	Timed Up and Go
CGA	Comprehensive Geriatric Assessment
MNA	Mini Nutritional Assessment
BMI	Body Mass Index
MoCA	Montreal Cognitive Assessment
BADL	Basic Activities of Daily Living
IADL	Lawton Instrumental Activities of Daily Living
